# Evaluation of the Flashtest multiplex qPCR system for rapid pre-laboratory screening for African swine fever infection

**DOI:** 10.2478/jvetres-2026-0022

**Published:** 2026-04-16

**Authors:** Marek Walczak, Łukasz Adaszek

**Affiliations:** 1 Department of Virology and Viral Animal Diseases, National Veterinary Research Institute, 24-100 Puławy, Poland; 2 Department of Epizootiology and Clinic of Infectious Diseases, University of Life Sciences, 20-612 Lublin, Poland

**Keywords:** ASF, ASFV, qPCR, diagnostics

## Abstract

**Introduction:**

African swine fever (ASF) is a devastating viral disease of domestic pigs and wild boar. As no safe vaccine is currently available, prevention relies primarily on strict biosecurity measures. Early diagnosis is essential to reduce the risk of ASF spread. Among the available methods, qPCR is considered the gold standard and is recommended by the World Organisation for Animal Health. The Flashtest system is designed to perform the complete workflow, from genetic material extraction to qPCR analysis, enabling rapid (under 1 hour) and accurate detection of various animal diseases in veterinary practice.

**Material and Methods:**

In this study, the Flashtest system was evaluated in ASF detection using blood samples collected during ASF outbreaks in 2025 in Poland and samples from experimentally infected pigs. Its performance was compared with a validated commercial qPCR assay employed by the National Reference Laboratory for ASF in Poland.

**Results:**

The system achieved up to 97% sensitivity, 100% specificity and reproducibility with a coefficient of variation below 3.5%. The limit of detection was estimated at 10^3^ TCID_50_/mL, and a slightly reduced amplification efficiency was observed.

**Conclusion:**

These results indicate that the Flashtest system reliably detects ASF in the majority of cases. Nevertheless, confirmatory testing is recommended for samples with low viral loads.

## Introduction

African swine fever (ASF) is a devastating viral disease of swine and wild boar, caused by the African swine fever virus (ASFV), a member of the Asfarviridae family. The ASFV genome consists of double-stranded DNA, ranges from 170 to 193 kbp in length and encodes more than 150 proteins (5). The disease is characterised by exceptionally high mortality rates, often approaching 100%. In the absence of an effective and safe vaccine, control strategies rely primarily on the depopulation of affected herds and biosecurity measures (6). Consequently, rapid diagnostic tools and stringent preventive measures remain essential for limiting the transmission and impact of ASF.

The qPCR represents the gold standard for ASF diagnosis and is the method recommended by the World Organisation for Animal Health (WOAH) (27). To date, multiple studies have reported the development of qPCR assays targeting various molecular markers, including the *b646l* gene (encoding the p72 protein), the *e183l* gene (encoding the p54 protein) and the *cp204l* gene (encoding the p30 protein) (12, 22, 23). Several authors have also reported multiplex qPCR methods that allow diagnosing and distinguishing ASFV genotypes or deletions within its genomes (9, 13, 30). These methods have demonstrated high sensitivity and reliability, which confirms their suitability for accurate diagnosis of ASF.

Flashtest is a system designed to perform the complete workflow from genetic material extraction to qPCR, enabling the detection of various animal diseases. It is intended for use in veterinary practice, providing rapid (under 1 hour) and accurate diagnosis. With ASF emerging as a significant concern, pre-laboratory diagnosis can accelerate the administrative processes involved in disease control and eradication. The Flashtest assay No. 3204 evaluated in this study targets *b646l*, commonly used in diagnostics and encoding the p72 protein as noted, and *ep402r* encoding the CD2v protein which is responsible for the haemadsorption phenomenon. It also targets unspecified gene within MGF region, which are the sites of multigene families involved in interferon modulation in the host and contributing to the virulence of ASFV (18, 24). The assay also enables differentiation between ASFV genotypes I and II. An endogenous control is included to help prevent diagnostic errors during sample preparation.

African swine fever virus exhibits tropism for myeloid lineage cells, particularly monocytes and macrophages abundant in blood (18). In a previous study, we demonstrated that blood represents one of the most reliable matrices for accurate diagnosis of ASF (25). The aim of this study was an evaluation of the Flashtest system for rapid pre-laboratory screening of ASF using blood samples collected during the ASF outbreaks in Poland in 2025 and samples from experimentally infected pigs. The test performance was compared with that of a validated commercial qPCR assay (Virotype) employed by the National Reference Laboratory for ASF in Poland.

## Material and Methods

### Viruses

To verify differentiation between ASFV genotypes I and II, the reference genotype I strain Ba71V (provided by the EU Reference Laboratory for ASF, Valdeolmos, Spain) was used propagated in a Vero cell culture, and a blood sample was used as the genotype II counterpart from a pig experimentally infected with the Arm07/CBM/c2 strain (Centro de Biología Molecular Severo Ochoa, Madrid, Spain). Specificity was assessed using porcine reproductive and respiratory syndrome virus 1 (Lelystad strain), classical swine fever virus (Alfort 187), border disease virus (Frijters), pseudorabies virus (Bartha), porcine circovirus type 2 (Stoon 1010) and swine influenza virus H1N1 (A/Sw/Pol/14131/2014).

### Samples

A total of 58 swine whole-blood samples were examined. The set of positive samples included blood without EDTA collected during ASF outbreaks in Poland in 2025 (n = 21) and blood with EDTA obtained from previous experiments carried out in pigs infected with the Arm07/CBM / c2 virulent genotype II ASFV strain (n = 11) (21, 25). The corresponding negative samples consisted of blood without EDTA (n = 11) and experimental blood without EDTA (n = 15). A total of 20 μL of each blood sample was mixed with 180 μL of PBS (Virotype assay) or sample preservation buffer (Flashtest assay) and subsequently subjected to DNA extraction.

### DNA extraction

For the Virotype assay, manual column-based DNA extraction was performed using the DNeasy Blood & Tissue Kit (Qiagen, Hilden, Germany), following the manufacturer’s protocol. For the Flashtest assay, automated magnetic bead-based DNA extraction was performed using the Flashtest extraction system, as recommended by the manufacturer.

### Real-time PCR

A real-time PCR was carried out with the Virotype ASFV PCR Kit 2.0 (Indical Bioscience, Leipzig, Germany) on a QuantStudio thermocycler (Thermo Fisher Scientific, Waltham, MA, USA) according to the manufacturer’s instructions. This was the validated test to which the Flashtest system was compared. The investigated real-time Flashtest ASFV Assay PCR No. 3204 was conducted on a Flashtest QPCRV1600 thermocycler in precision mode, following the manufacturer’s instructions (Flashtest Bio, Wuxi, China).

### Sensitivity, limit of detection, reproducibility and linearity

Diagnostic sensitivity was determined as the proportion of true positives among all laboratory-confirmed (Virotype assay) positive samples. Ninetyfive percent confidence intervals for the sensitivity estimates were calculated using the Wilson score method without continuity correction. Analytical sensitivity was evaluated using 10-fold serial dilutions of a reference titrated ASFV-positive blood sample. The limit of detection (LOD) was defined as the lowest dilution at which all 10 replicates (10/10) yielded a positive result. Reproducibility was evaluated across three independent runs of a reference blood sample diluted into three concentration ranges, with each run subjected to three separate extractions. Variability was quantified using the coefficient of variation, defined as the ratio of the SD to the mean. Amplification efficiency was assessed using a standard curve based on 12 data points from ten-fold serial dilutions prepared from ASFV-positive, titrated (10^6^ TCID_50_/mL) whole blood. Each dilution was analysed in triplicate to determine threshold cycle (Ct) values. These were plotted against the virus titre.

### Statistical analysis

Linear regression analysis was performed to obtain the standard curve, including slope, y-intercept and correlation coefficient (R^2^). The Wilcoxon signed-rank test was used to compare the consistency between the Flashtest and reference Virotype assays. Statistical analyses were performed in GraphPad Prism 8.4.3 (GraphPad Software, San Diego, CA, USA).

## Results

### Differentiation of ASFV genotypes I and II

The system accurately differentiated ASFV genotypes I and II. Amplification of the reference strain Ba71V occurred only partially: the p72 target was replicated, while the CD2v and MGF targets were not. This corresponded to the known properties of this strain (lack of haemadsorption and presence of only limited MGF genes, of which none is targeted by the assay) (1, 17). In the blood sample collected from a pig experimentally infected with a genotype-II strain of ASFV, amplification of all three targets was observed. The corresponding amplification curves are shown in Supplementary Fig. S1.

### Sensitivity, limit of detection and specificity of the Flashtest

In the group of positive samples confirmed in the laboratory with the Virotype assay (n = 32), one sample was found negative, and two additional samples showed detectable amplification only of CD2v, and none of p72 or MGF. These false-negative samples had Ct values ranging from 34.5 to 36.4 in the Virotype assay, corresponding to low viral loads (1.5–5 × 10^2^ TCID_50_/mL). These are below the LOD of the Flashtest, which is estimated at 10^3^ TCID_50_/mL. No amplification was detected in the negative samples (n = 26) or in the reference samples for PRRSV, CSFV, BDV, PRV, PCV2 or SIV, indicating 100% specificity. The sensitivity of the Flashtest assay for each target is presented in Table 1.

**Table 1.** Sensitivity of the Flashtest multiplex qPCR assay for p72 (*b646L*) CD2v (*ep402R*) and an MGF targets

Target	Number of TP	Number of FN	Sensitivity (95% CI)
p72	29	3*	90.6% (75.8–96.8)
CD2v	31	1*	97.0% (84.7–99.5)
MGF	29	3*	90.6% (75.8–96.8)

1 95% CI – 95% confidence interval; TP – true positives; FN – false negatives;

* – samples below estimated limit of detection

### Comparison of Ct values between assays

Paired analysis using the Wilcoxon signed-rank test revealed no statistically significant differences between the Flashtest and Virotype assays for the p72, CD2v or MGF genes. More consistent results were obtained for CD2v and MGF, while for p72 the difference between the Flashtest Ct and the Virotype Ct nearly approached the statistical significance threshold (P–value = 0.0506), because of higher deviations. The median difference ranged from –0.54 to 0.58 between the targets. The Ct values showed strong correlations between the assays (Spearman’s r_s_ = 0.95–0.96; P–value < 0.0001), indicating high concordance and preservation of measurement trends. The results are summarised in Table 2, and the raw results are presented in Supplementary Table S1.

**Table 2.** Paired analysis of threshold cycle (Ct) values for the p72 (*b646L*) CD2v (*ep402R*) and an MGF targets, amplified using the Flashtest (Flash) assay in comparison with the Virotype (Viro) qPCR assay

Target	Median difference in Ct (Flash/Viro)	Wilcoxon p	Spearman r_s_
p72	0.58	0.0506	0.9522
CD2v	–0.24	0.8962	0.9633
MGF	–0.54	0.6352	0.9527

### Reproducibility

The reproducibility of the Flashtest assay was evaluated in high, medium and low viral load sample concentrations. For all three genes, mean Ct values increased as the amount of genetic material decreased, as expected. Standard deviations ranged from 0.30 to 1.07 cycles, and coefficients of variation were below 3.5% across all ranges. Slightly higher variability was observed at the lowest template concentrations, reflecting stochastic effects inherent to low genetic material concentration. Overall, all three targets demonstrated consistent performance across their respective dynamic ranges, making the assay suitable for qualitative analysis.

**Table 3.** Reproducibility parameters Flashtest multiplex qPCR assay

Target	Range (TCID_50_/mL)	Mean Ct(±SD)	CV (%)
p72	High (10^6^)	20.97 (0.56)	2.65
	Mid (10^4^)	27.76 (0.54)	1.96
	Low (10^3^)	32.02 (1.07)	3.34
CD2v	High (10^6^)	20.09 (0.39)	1.96
	Mid (10^4^)	26.76 (0.40)	1.50
	Low (10^3^)	30.88 (1.02)	3.30
MGF	High (10^6^)	19.96 (0.30)	1.51
	Mid (10^4^)	26.81 (0.38)	1.41
	Low (10^3^)	30.86 (0.82)	2.67

1 TCID_50_ –50% tissue culture infective dose; Ct – threshold cycle; CV – coefficient of variation

### Linearity of the Flashtest assay

**Fig. 1.**
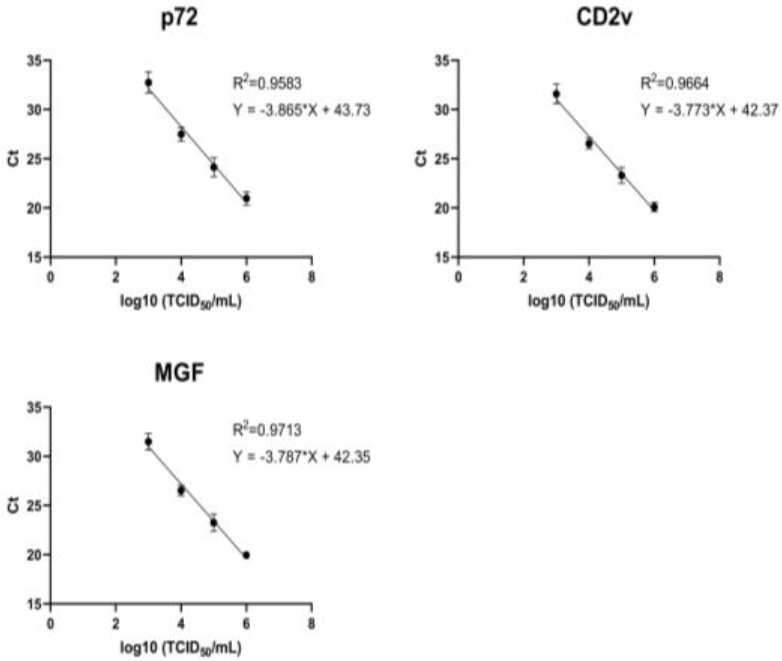
Linearity parameters of the Flashtest multiplex qPCR assay. Ct – threshold cycle; TCID_50_ – 50% tissue culture infective dose. Error bars indicate SD

The assay demonstrated a linear correlation between the Ct values and the logarithm of the virus titre in whole blood positive for ASFV for all three targets. The coefficients of determination (R^2^) were 0.9583 for p72, 0.9664 for CD2v and 0.9713 for MGF. Slight deviations from linearity were observed at the lowest viral concentrations. The regression slopes ranged from –3.865 to –3.773, corresponding to amplification efficiencies of approximately 82–84%, which were below the optimal range of 90–110%. All regressions were highly significant (P-value < 0.0001), indicating that the relationships between Ct values and viral genome quantity were statistically robust. Linearity for all three targets is illustrated in Fig. 1.

## Discussion

Control of ASF depends heavily on rapid and reliable diagnosis to prevent further spread. Laboratories with experienced scientists are proficient in ASF detection, but do not offer the most rapid results because transport from sampling site to laboratory takes some time. Numerous alternative methods have been explored for on-site use, including loop-mediated isothermal amplification, lateral flow devices, recombinase polymerase amplification, CRISPR-based assays, cross-priming amplification assays and miniaturised sensors based on surface plasmon resonance (SPR) (3, 4, 8, 11, 14, 17, 28). Each of these approaches, however, has inherent limitations, including the risk of false-positive results, low sensitivity or susceptibility to misinterpretation. The real-time PCR, the output of which was traditionally the preserve of specialised laboratories, is now becoming more widely accessible (15). It is a well-established and reliable technique, and its use as a commissioned diagnostic test in routine veterinary practice could provide substantial benefits for disease monitoring and animal health protection.

The Flashtest system, specifically designed for veterinary practice, is based on qPCR technology. It combines a broad diagnostic range for both companion and livestock animals with user-friendly operation. The system integrates automatic magnetic bead–based nucleic acid extraction, which enhances reproducibility and reduces the risk of operator error. An important advantage of Flashtest in ASF diagnostics is its multiplexed targeting approach. Similar multiplex assays have previously been described by Yang *et al*. (28) and Lin *et al*. (13) and were further recommended by the WOAH (26). The multiplexed approach not only increases the probability of detection in cases where amplification of one of the genes fails – thereby reducing the risk of false-negative results – but also enables the identification of strains with specific epidemiological relevance. These include non-haemadsorbing strains lacking the CD2v gene and strains with deletions in MGFs that influence viral virulence (11, 12). Furthermore, the system discriminates between genotypes I and II. Although genotype II has been present in Europe since 2007 and remains dominant in the region, the risk of genotype I introduction is increasing. In 2021, genotype I ASFV was reported in China (21), and a genotype I/II recombinant resistant to ASF vaccines emerged shortly after (29), which has also been detected in Russia (10). The introduction of genotype I into previously unaffected regions would pose significant epidemiological risks. In this context, the ability to differentiate ASFV genotypes is of particular importance.

However, certain limitations of the Flashtest should be noted, including a relatively high detection limit and a suboptimal amplification efficiency, as indicated by the linearity of the assay. Importantly, unlike many preliminary evaluations, this study employed clinical blood samples (also for the linearity assay), which more accurately reflect the practical challenges encountered in routine testing. Such samples inherently contain naturally occurring factors that can influence assay performance – including PCR inhibitors, variable viral loads and other matrix-related effects – and they provide a means of assessing the Flashtest’s reliability under conditions like those in the field (19). It should be noted that the higher LOD observed in this study is partly due to the smaller blood volume used – 20 μL instead of the 200 μL typically used in laboratory-based DNA-extraction assays. As a result, the LOD of the Flashtest is approximately ten times higher than that of standard qPCR assays such as the Virotype PCR. However, the 1 : 10 sample dilution is recommended by the Flashtest manufacturer, and the system has been specifically optimised to function under these sample volume and dilution conditions. The linearity results demonstrated that the Flashtest provides consistent amplification across the three viral targets, while showing minor deviations from the ideal linearity expected in perfectly optimised qPCR reactions. The suboptimal efficiency (82–84%) and slightly reduced correlation coefficient observed when testing positive blood samples primarily affect the precision of quantitative estimates, particularly at low template concentrations. However, these parameters do not compromise the qualitative interpretation of results (positive/negative), as the assay reliably detects amplification in ASFV-positive samples. The main consequence is a moderately increased detection threshold, which should be considered when interpreting low viral load samples (2, 7). It is worth noting the physical limitations of the Flashtest system. Its small size limits sample throughput and permits up to four samples in the extraction module and up to sixteen in the thermocycler simultaneously. Such throughput would represent a considerable constraint in a professional laboratory setting. Nevertheless, since the device is intended for diagnostic use in veterinary practice, this limitation does not preclude its practice-based diagnostic applicability.

## Conclusion

Flashtest demonstrated its suitability for rapid pre-laboratory ASF screening, providing diagnostic performance sufficient to generate reliable results; however, in some cases, confirmatory testing is recommended for samples with low viral loads.

## Supplementary Material

Supplementary Material Details

Supplementary Material Details
