# Supplementary material for: Evaluation of the Flashtest multiplex qPCR system for rapid pre-laboratory screening for African swine fever infection
Source: J Vet Res. 2026 Apr 16;70(2):185–90. doi: 10.2478/jvetres-2026-0022 (PMC13334293; doi:10.2478/jvetres-2026-0022)
Supplement: Supplementary file 1 — Supplementary Material Details [file jvetres-2026-0022_sm.pdf]

A

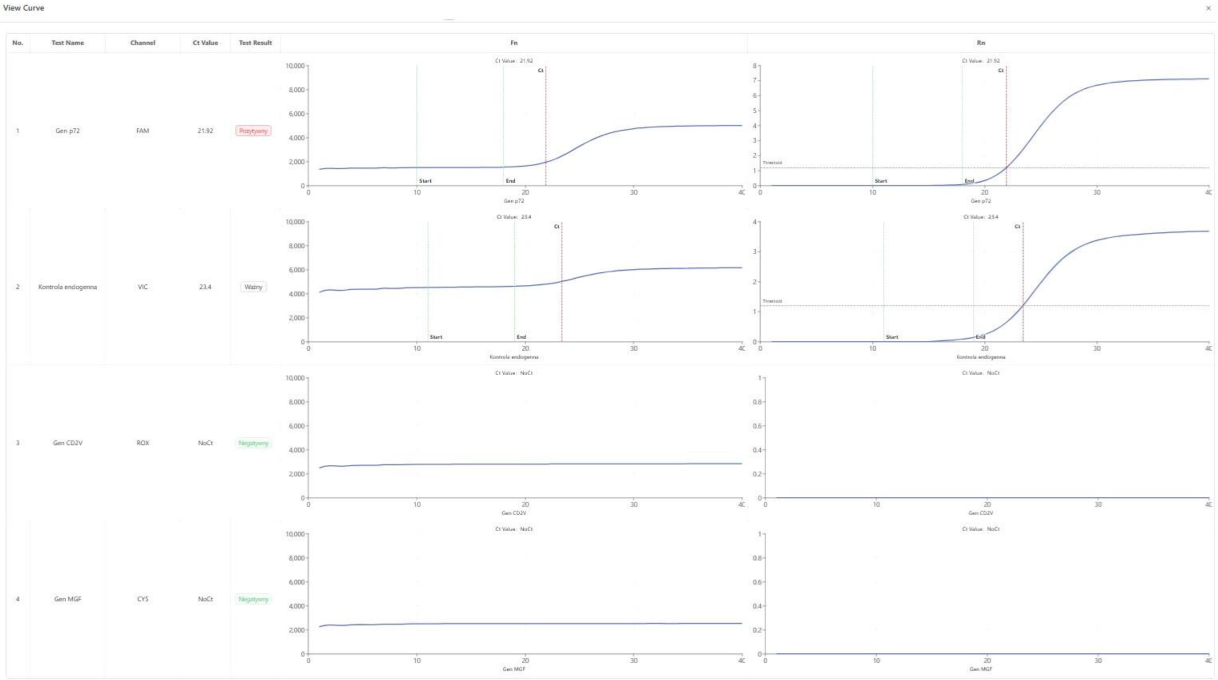

B

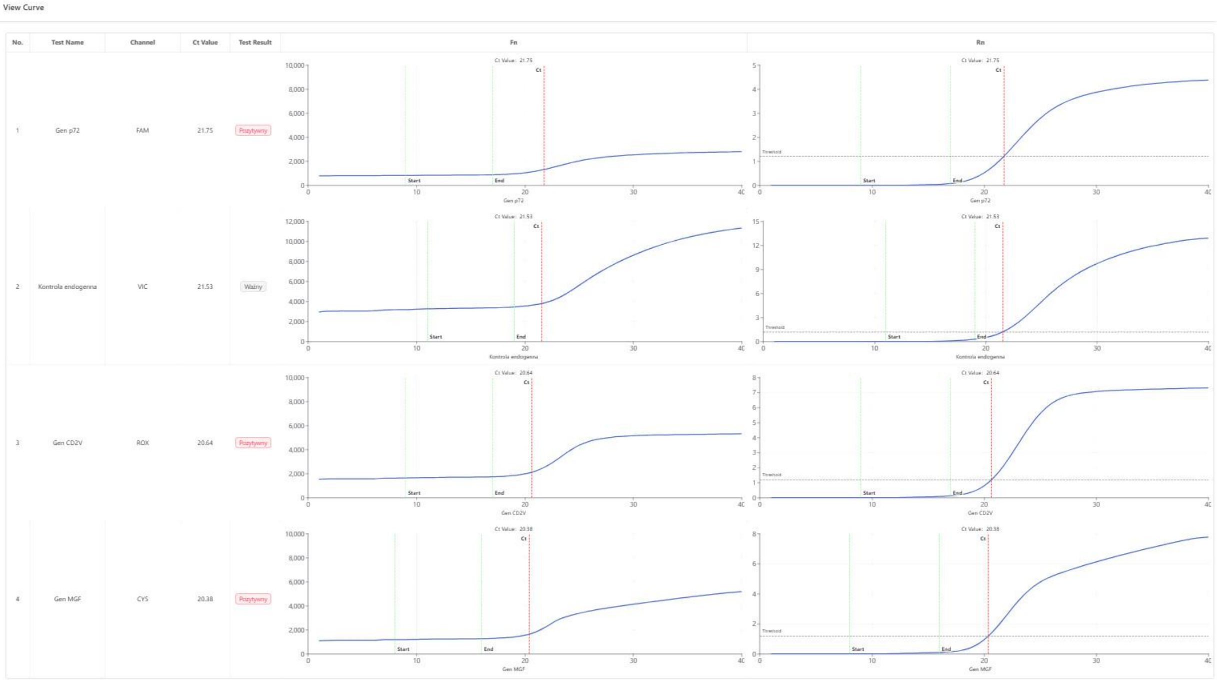

Supplementary Fig. S1. Multiplex Flashtest qPCR for target-specific amplification of the p72 (*b646L*), CD2v (*ep402R*), and MGF targets along with the assay's endogenous control, in Ba71V reference strain (A) and Arm07/CBM/c2 genotype II-infected pig blood (B)

FAM – 6-carboxyfluorescein; VIC – 2′-chloro-7′phenyl-1,4-dichloro-6-carboxyfluorescein; ROX – 6-carboxy-X-rhodamine; CY5 – cyanine 5
