# Supplementary material for: Evaluation of the Flashtest multiplex qPCR system for rapid pre-laboratory screening for African swine fever infection
Source: J Vet Res. 2026 Apr 16;70(2):185–90. doi: 10.2478/jvetres-2026-0022 (PMC13334293; doi:10.2478/jvetres-2026-0022)
Supplement: Supplementary file 2 — Supplementary Material Details [file jvetres-2026-0022_sm1.pdf]

Supplementary Table S1. Cycle threshold (Ct) values for Virotype and Multiplex Flashtest qPCR, showing target-specific amplification of p72 (*b646L*), CD2v (*ep402R*), and MGF targets, along with the assay's endogenous control (ENDCtrl)

Sample No.	Sample ID	Virotype assay (Ct)	Flashtest assay (Ct)			
			p72	CD2v	MGF	ENDCtrl
1	26463-3/25	21.14	20.18	20.61	20.6	20.56
2	27475-31/25	21.74	20.79	20.03	20.5	20.06
3	27475-32/25	20.88	21.15	20.01	19.92	20.52
4	264612-14/25	25.11	25.31	24.31	24.35	25.74
5	26462-19/25	25.31	23.55	22.84	22.85	22.89
6	26462-20/25	24.57	23.65	22.83	22.72	23.1
7	27670-1/25	19.88	19.17	19.01	18.8	18.08
8	27672-3/25	26.65	26.39	25.37	25.02	21.64
9	27672-5/25	35.37	no C_t	no C_t	no C_t	24.53
10	27672-8/25	34.49	no C_t	36.74	no C_t	23.48
11	27672-10/25	31.95	33.01	31.66	31.43	23.95
12	27672-11/25	33.52	37.43	34.23	35.94	25.19
13	27672-13/25	32.84	32.27	31.39	30.63	25.89
14	27672-21/25	21.54	22.91	22.1	21.79	20.17
15	24858-5/25	25.79	25.11	24.26	24.16	24.13
16	24858-6/25	20.31	20.00	20.47	20.29	20.00
17	24858-13/25	24.399	24.46	23.36	23.01	23.57
18	30358-14/25	31.98	32.7	31.74	31.03	23.37
19	30358-15/25	36.39	no C_t	38.88	no C_t	23.12
20	30207-10/25	32.69	37.08	34.06	36.64	24.19
21	30207-19/25	32.85	35.38	33.38	35.61	24.26
22	F19	28.70	32.96	31.36	31.24	26.71
23	J.2	29.32	31.86	30.7	30.47	25.99
24	I.3	22.41	24.64	23.7	23.66	24.19
25	H.5	20.57	18.82	17.9	17.92	18.87
26	C.8	19.31	16.47	15.59	15.56	16.76
27	C.7	24.96	28.86	27.61	27.32	25.79
28	L.4	28.67	30.2	29.07	28.49	23.82
29	G.5	21.17	21.75	20.64	20.38	21.53
30	G.25	27.9	29.16	27.84	27.66	24.46
31	H.25	25.57	29.65	28.13	27.91	25.21
32	I.25	23.85	27.1	25.83	25.56	25.92

1–21 – nonEDTA samples (outbreak); 22–32 – EDTA samples (experimental)
